# Consensus recommendations for measuring the impact of contraception on the menstrual cycle in contraceptive clinical trials

**DOI:** 10.1016/j.contraception.2025.110829

**Published:** 2025-01-27

**Authors:** Amelia C.L. Mackenzie, Stephanie Chung, Emily Hoppes, Nora Miller, Anne E. Burke, Sharon L. Achilles, C. Leigh Allen, Luis Bahamondes, Diana L. Blithe, Vivian Brache, Rebecca L. Callahan, Alice F. Cartwright, Kathryn B.H. Clancy, Enrico Colli, Amanda Cordova-Gomez, Elizabeth C. Costenbader, Mitchell D. Creinin, Hilary O.D. Critchley, Gustavo F. Doncel, Laneta J. Dorflinger, Alison Edelman, Thomas Faustmann, Christoph Gerlinger, Lisa B. Haddad, Julie Hennegan, Cássia Raquel Teatin Juliato, Simon P.S. Kibira, Diana Mansour, Andres Martinez, Kristen A. Matteson, Jacqueline A. Maybin, Alexandria K. Mickler, Kavita Nanda, Chukwuemeka E. Nwachukwu, Funmilola M. OlaOlorun, Kevin J. Peine, Chelsea B. Polis, Carolina Sales Vieira, Regine Sitruk-Ware, Jennifer A. Smit, Marsden Solomon, Lisa M. Soule, Douglas Taylor, Elizabeth E. Tolley, Olivia Vandeputte

**Affiliations:** a Global Health and Population, FHI 360, Washington, DC, United States; b Global Health and Population, FHI 360, Durham, NC, United States; c Department of Maternal and Child Health, University of North Carolina Gillings School of Global Public Health, Chapel Hill, NC, United States; d Mann Global Health, Waterbury, VT, United States; e Johns Hopkins University School of Medicine and Johns Hopkins Bloomberg School of Public Health, Baltimore, MD, United States; f Bill & Melinda Gates Foundation, Seattle, WA, United States; g Eunice Kennedy Shriver National Institute of Child Health and Human Development (NICHD), National Institutes of Health (NIH), Bethesda, MD, United States; h Department of Obstetrics and Gynecology, University of Campinas, Faculty of Medical Sciences, Campinas, São Paulo, Brazil; i Profamilia, Santo Domingo, Dominican Republic; j University of Illinois Urbana-Champaign, Urbana, IL, United States; k Exeltis R&D, Madrid, Spain; l Research, Technology, and Utilization Division, Office of Population and Reproductive Health, Bureau for Global Health, United States Agency for International Development and the Public Health Institute, Washington, DC, United States; m University of California, Davis, Sacramento, CA, United States; n Centre for Reproductive Health, Institute for Regeneration and Repair, University of Edinburgh, Edinburgh, Scotland, UK; o CONRAD – Eastern Virginia Medical School, Norfolk, VA, United States; p Oregon Health and Science University, Portland, OR, United States; q Bayer AG, Berlin, Germany; r Universität des Saarlandes, Homburg, Saarland, Germany; s Center for Biomedical Research, Population Council, New York, NY, United States; t Maternal, Child and Adolescent Health Program, Burnet Institute, Melbourne, Victoria, Australia; u Makerere University School of Public Health, Kampala, Uganda; v Women’s Health Directorate, Newcastle upon Tyne Hospitals NHS Foundation Trust, Newcastle upon Tyne, England, UK; w Global Health and Population, FHI 360, Atlanta, GA, United States; x University of Massachusetts Chan Medical School, Worcester, MA, United States; y College of Medicine, University of Ibadan, Ibadan, Oyo, Nigeria; z Department of Obstetrics and Gynecology, Ribeirao Preto Medical School, University of São Paulo, Ribeirao Preto, São Paulo, Brazil; aa Wits MatCH Research Unit, Department of Obstetrics and Gyanaecology, The University of the Witwatersrand, Durban, KwaZulu-Natal, South Africa; ab FHI 360, Nairobi, Kenya; ac Independent Consultant, Bethesda, MD, United States; ad MSI Reproductive Choices, London, England, UK

**Keywords:** Bleeding patterns, Consensus, Contraceptive clinical trials, Contraceptive-induced menstrual changes, Menstrual cycle, Patient reported outcome measures

## Abstract

**Objective:**

We sought to develop consensus recommendations for measurement and analysis of data on contraceptive-induced menstrual changes (CIMCs) in contraceptive clinical trials. We built upon previous standardization efforts over the last 50 years and prioritized input from a variety of global experts and current regulatory authority guidance on patient-reported outcomes.

**Study design:**

We completed a formal consensus-building process with an interdisciplinary group of 57 experts from 30 organizations and 14 countries in five global regions who work across academia, nonprofit research organizations, the pharmaceutical industry, and funding agencies. Smaller topical working groups drafted and revised recommendations.

**Results:**

We developed 44 consensus recommendations, including research approaches to establish the evidence for future improvement in the measurement and analysis of CIMC data and guidance for investigators to implement presently. Priority recommendations call for simplification of terminology to make measurement accessible and patient-centered, accounting for intrinsic and extrinsic factors that may impact outcomes during study design and recruitment, standardized data collection of primary CIMC and acceptability outcomes, and harmonized approaches for analysis of these data, including addressing missing data.

**Conclusion:**

By virtually convening a large group of global experts working across disciplines and sectors via a formal methodology, we developed consensus recommendations that will improve the current and future measurement and analysis of CIMC data in contraceptive clinical trials. Using these standardized approaches will permit valid and reliable contraceptive product labeling on CIMC outcomes that matter to users and greater comparability across trials that can inform clinical guidance and contraceptive counseling.

**Implications:**

Consensus recommendations on measuring bleeding changes and related outcomes in contraceptive clinical trials can improve reporting of standardized, patient-centered outcomes in future product labeling. These improvements can enable healthcare providers to offer more relevant guidance on contraceptives and users to make more informed decisions about their choice of method.

## 1. Introduction

**Fig. 1.**
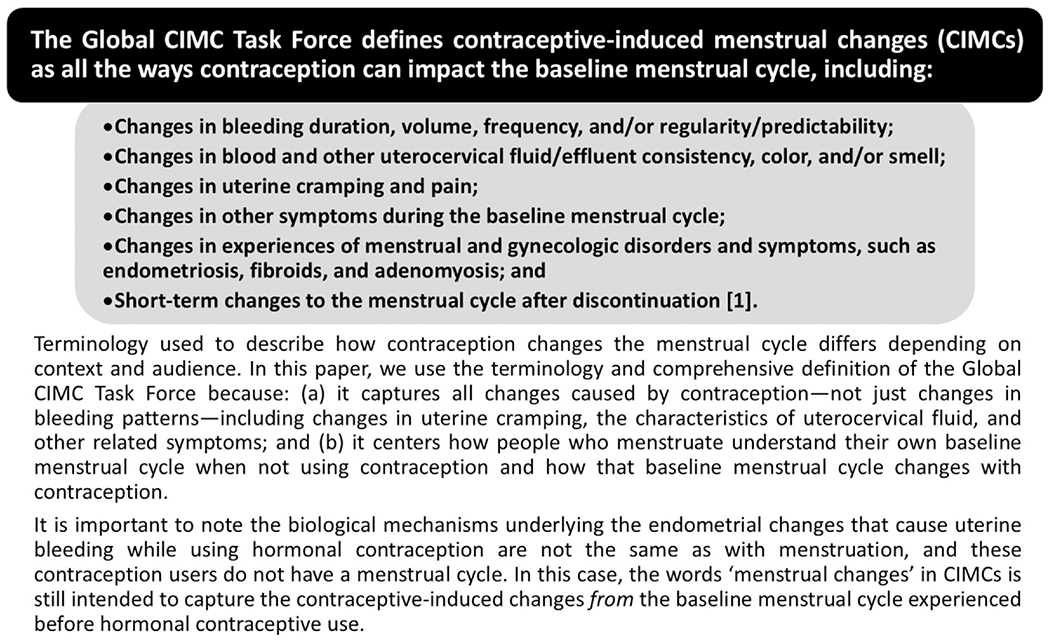
Definition and terminology of contraceptive-induced menstrual changes (CIMCs).

Contraceptive use can cause changes in uterine bleeding patterns, uterocervical fluid, and uterine cramping and can affect how users experience menstrual and gynecologic disorders and symptoms. Collectively, we refer to these changes as contraceptive-induced menstrual changes (CIMCs) (Fig. 1). Experiencing CIMCs can both negatively and positively influence the use dynamics, health, and wellbeing of contraceptive users, as well as the individual and sociocultural acceptability of contraception [1]. When users find CIMCs undesirable, negative consequences can include contraceptive dissatisfaction, reduced quality of life, increased burden managing menstrual health, and reduced sexual wellness [1–4]. Undesired CIMCs can also result in potential unintended pregnancy if users discontinue or switch to less effective contraception while still wanting to prevent pregnancy. On the other hand, CIMCs users consider desirable can result in benefits, such as management of menstrual and gynecologic disorders and symptoms, method satisfaction, improved sexual wellness, and reduced burden or costs of menstrual materials [1–6]. Which CIMCs users find desirable or undesirable can vary widely, influenced by individual preferences and community-level social norms—especially around menstruation, menstrual health, and sexual and reproductive health—as well as perspectives of partners and wider social and contextual factors [1–3,7,8]. Previous research suggests users often prefer reduced bleeding and cramping, and dislike increased bleeding and unpredictability [1,2]. Considerations regarding CIMCs have played a notable role in contraceptive development decisions since the preclinical and clinical research for the first oral contraceptive in the 1950s, and these decisions still influence the current contraceptive method mix and contraceptive development today [9–11]. There have been several endeavors to standardize the measurement and reporting of CIMC data in contraceptive clinical research, which Creinin and colleagues [12] recently reviewed.

Current approaches for measuring CIMCs in contraceptive clinical trials continue to have limitations, including burdensome data collection, a lack of uniform definitions provided to trial participants to understand key terms, and the use of patient-reported outcome data without evidence of validation in line with current regulatory guidance on patient-focused drug development [13–16]. These limitations are particularly relevant for standard measurement of CIMCs in trials globally because—for topics like menstruation, with high levels of stigma—there are generally low levels of shared understanding and common references within and between communities and contexts [8,17,18]. In addition, how CIMC outcomes are currently reported can be difficult for providers to use in contraceptive counseling of new methods and are often not meaningful to contraceptive users to facilitate their informed method decision-making. Building upon previous standardization efforts—and in collaboration with many of the experts who contributed to this earlier work—we sought to address these limitations by developing consensus recommendations to further improve the measurement of CIMC data in contraceptive clinical trials now and in the future.

## 2. Methods

### 2.1. Consensus-building methodology

**Fig. 2.**
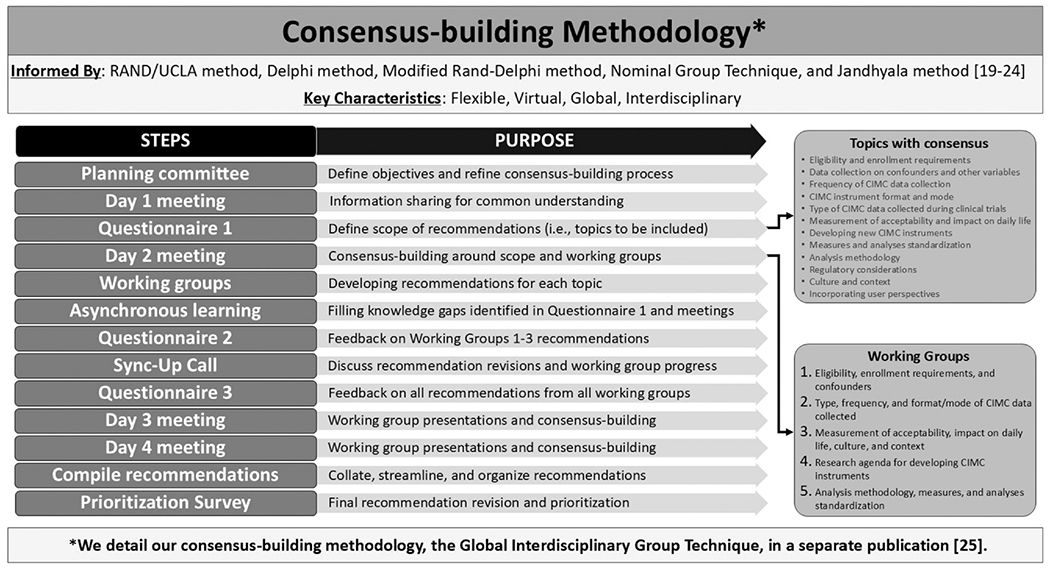
Consensus-building methodology for developing recommendations on measuring and analyzing contraceptive-induced menstrual change (CIMC) data in contraceptive clinical trials, 2023.

Our consensus-building approach prioritized: (1) incorporating a variety of interdisciplinary and global perspectives, (2) considering current guidance from the US Food and Drug Administration and other regulatory authorities around patient-reported outcomes, and (3) including all types of CIMCs, not just changes in bleeding patterns, to align with the Global CIMC Task Force’s comprehensive definition (Fig. 1) [1]. We used an amalgamation of the RAND/UCLA Appropriateness Method, the Delphi method, the Modified Rand-Delphi method, the Nominal Group Technique, and the Jandhyala method because no single approach met all our needs [19–24]. We present additional details on our adapted, new methodology, the Global Interdisciplinary Group Technique, in a separate publication [25], but briefly, we completed a fully virtual consensus-building process, including four half-day meetings for all experts to join, recommendation development within five smaller topical working groups, and a series of consensus questionnaires around the scope and details of the recommendations (Fig. 2; Appendix Fig. A.1). Each questionnaire asked working group members about their agreement/disagreement on a four-point Likert-like scale (i.e., strongly agree, somewhat agree, somewhat disagree, strongly disagree), and we decided recommendations needed at least 75% agreement to be included in our consensus list. Questionnaires also included at least one open-ended question for any additional input from working group members, and one author (SC) analyzed these results. The Office of International Research Ethics at FHI 360 determined the project protocol was research that was exempt from Institutional Review Board review. Experts participating in the consensus-building process provided digital written consent.

### 2.2. Expert characteristics

A total of 57 experts participated in our consultation and consensus-building process. They were from 30 organizations—across academia, nonprofit research organizations, funding agencies, and the pharmaceutical industry—and 14 countries in five global regions (i.e., Northern America, Europe, Africa, Latin America & the Caribbean, and Oceania, listed in order of frequency). Of those experts, 44 self-selected into working groups to develop the recommendations. Working group members represented over 675 combined years of experience in contraceptive clinical trials, other contraceptive research and development, menstrual cycle and menstrual health research, social-behavioral research, contraceptive prescribing, and/or other relevant fields (Table 1). Almost all working group members (86%) reported having either experienced CIMCs themselves or observed someone personally close to them experiencing CIMCs.

## 3. Results

### 3.1. Consensus recommendations

We developed 44 consensus recommendations, nearly half of which reached greater than 90% consensus (see Appendix Table A.1 for consensus details). We organized the recommendations under six section headings: (1) standardization; (2) instruments for measuring CIMCs; (3) trial design, protocol development, and participant recruitment; (4) data collection; (5) data analysis; and (6) areas for exploratory research (Table 2). Our recommendations include research approaches to establish the evidence for future improvement in the measurement of CIMC data and guidance for investigators to implement in the meantime. Because we aimed to build upon previous standardization efforts for CIMCs in clinical trials, our recommendations often incorporate concepts from previous criteria, especially the work of Creinin and colleagues [12]. Recommendations 1.3 and 5.2 draw heavily from these most recent criteria. Several recommendations also incorporate regulatory authority guidance and established research approaches for patient-reported outcomes like CIMCs.

Our recommendations begin in Section 1 of Table 2 by describing standardization approaches and their rationale, noting the need to ensure comparability to previous approaches, as well as some contextual flexibility. This first section is also where we first note additional research will be necessary to align with many of our recommendations, so throughout, we provide direction on approaches to implement presently, while that research is conducted (i.e., recommendations beginning with “Presently…”). In line with that additional research, we call for new instrument development in Section 2 based on a recent systematic review [55] and detail what critical and ideal elements those new instruments must include. The recommendations in Section 2 are also the first to refer to regulatory guidance and best practices around developing and validating instruments for patient-reported outcomes.

In Section 3, we explain important considerations when designing contraceptive clinical trials, including confounders and other intrinsic and extrinsic factors related to CIMCs, baseline data collection, input from researchers and Community Advisory Boards at trial sites, the use of diverse trial sites in later trials, and provider training. We list, in Section 4, the data on CIMCs that should presently be collected while the research to develop improved instruments described in the previous sections is conducted. In Section 5, we then specify how researchers should analyze these data, including recommended variables, descriptive statistics, inferential statistics, sensitivity analyses, handling of missing data, and data visualization. We end with two recommendations in Section 6 on considerations for advancing measurement and analysis of CIMC data in the future, which complement and extend a previous CIMC research agenda developed by the Global CIMC Task Force [1].

### 3.2. Priorities

We assessed the priority of recommendations based on their urgency and the potential influence of their implementation (see Appendix Table A.1 for priority details). Over 85% of experts deemed seven recommendations as a priority: recommendations 1.1, 2.2, 4.1, 4.11, 5.2, 5.3, and 5.11, indicated by dark shading in Table 2. Recommendation 1.1 calls for standardization and simplification of terminology. In support of this, recommendation 2.2 outlines best practices for making both terminology and measurement instruments simple, accessible, and patient-centered. Also aligning with recommendation 1.1 is recommendation 5.2, which lays out important terminology and definitions for use across trials, defining a bleeding episode, as well as duration, frequency, and volume of bleeding episodes. Recommendation 1.1 also calls for standardization in the type of CIMC data collected, and recommendations 4.1 and 4.11 provide more detail on data collection, with recommendation 4.1 covering the primary outcomes of CIMCs—including bleeding and spotting days and frequency, duration, and volume of bleeding episodes, and recommendation 4.11 outlining the data on acceptability and quality of life. Recommendations 5.3 and 5.11 outline key variables and details on missing data we recommend researchers report for all contraceptive clinical trials.

In addition to these seven recommendations, over 75% of experts deemed 12 other recommendations as a priority, indicated by lighter shading in Table 2. These additional priorities are recommendations 1.3 on the definition of spotting and bleeding, 2.1 on the need for new instruments, 3.2 on including participants with a range of body mass indexes, 3.3 on handling intrinsic and extrinsic factors that may influence CIMCs, 3.7 on incorporating additional CIMC data in later trials, 4.3 and 4.4 on recommended data collected by trial phase, 4.6 on using validated measures for bleeding volume for trials where this is an outcome for labeling purposes, 4.9 on use of electronic data collection for diaries, 5.4 on descriptive statistics for the number of bleeding and spotting days, 5.10 on sensitivity analyses, and 5.14 on open data.

## 4. Discussion

### 4.1. How our recommendations differ from previous standardization criteria

**Fig. 3.**
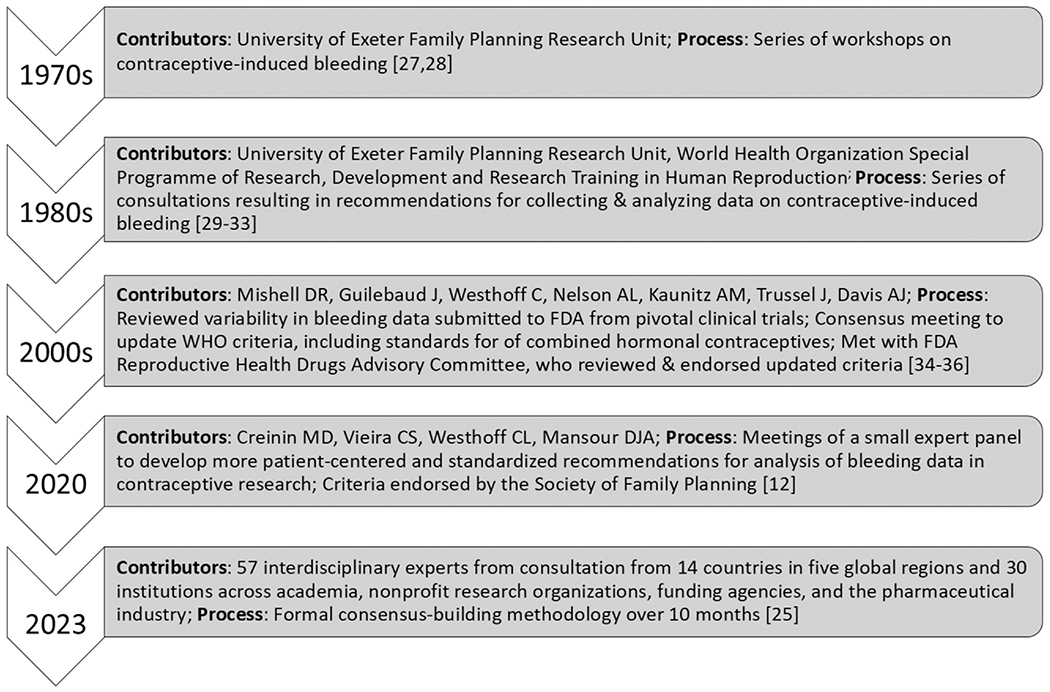
Methodology of efforts to standardize the measurement and analysis of contraceptive-induced menstrual change (CIMC) data in contraceptive clinical trials.

We developed consensus recommendations to improve the current and future measurement and analysis of CIMC data in contraceptive clinical trials to address persisting limitations in current approaches and incorporate current regulatory authority guidance on patient-reported outcomes. These recommendations build upon previous approaches at standardization around CIMCs over the past 50 years [12,56–65]. However, they differ in important ways, particularly related to our consensus-building methodology, which has affected the content and breadth of our recommendations (Fig. 3).

Although any consensus-building process will inevitably reflect the perspectives and priorities of those involved, by virtually convening an interdisciplinary group of over 50 experts from around the world, our recommendations echo those diverse perspectives and experiences. Most notably, this is the first time recommendations have included uterine cramping and pain, a previously overlooked but important outcome for people who menstruate. By beginning with the Global CIMC Task Force’s expanded definition of CIMCs and including experts in menstrual health, heavy menstrual bleeding, patient-reported outcomes, and social-behavioral research, we have extended the scope of these recommendations by considering all CIMCs, not just changes in bleeding patterns.

Our interdisciplinary group also had the expertise to incorporate recommendations on the measurement of acceptability outcomes and to integrate best practices from other fields. For example, we borrow from a practice common in clinical trials for prevention and treatment of HIV and other sexual and reproductive health disciplines by proposing engagement with Community Advisory Boards at trial sites. Our recommendations also use inclusive language throughout, support more diversity in trial sites and trial participants to better reflect the population of future contraceptive users and improve external generalizability, and suggest collecting data on information related to gender identity and sexual orientation. For sponsors and investigators interested in additional guidance on including transgender, gender diverse, and nonbinary participants in contraceptive research, we suggest the recent work of Moseson and colleagues [66] on sexual and reproductive health research more broadly and Sims and Appenroth [67] focused on contraceptive development.

Overall, our methodology sought to balance the perspectives and priorities of all experts in the consensus-building process and all stakeholders in the contraceptive clinical trial ecosystem, including trial investigators and other researchers, trial sponsors and funders, regulators, trial participants, and the future providers and users of the new contraceptives being developed in trials. We propose our consensus-building methodology, the Global Interdisciplinary Group Technique, as a model—for the field of contraception, wider sexual and reproductive health, and beyond—of how to successfully bring a large, interdisciplinary, and diverse group of people together from around the world to come to consensus on a topic that had previously eluded such agreement [25].

### 4.2. Areas of disagreement during consensus-building

Although we ultimately reached consensus on all recommendations, we encountered some recurring areas of disagreement that likely warrant further attention within the field of contraceptive research. In some instances, differences occurred simply due to the interdisciplinary and global nature of our expert group and the distinct perspectives that inherently arise when convening those with different trainings, experiences, and areas of expertise. Often these differences also emerged between those approaching the work from a biomedical perspective and those working within social-behavioral research. These areas of contention and ensuing conversations, however, were essential to thoroughly address a topic as multifaceted as measuring CIMCs in the context of clinical trials. As contraceptive research aims to incorporate more diverse and interdisciplinary perspectives to tackle increasingly complex, global problems, we can expect opportunities to embrace such differences to become more common.

As an illustrative example, some areas of disagreement could be distilled down to differing perspectives along a continuum. On one end of the spectrum was a preference to recommend modest improvements to current approaches that would be feasible and straightforward to implement, aligning with past and present norms and existing systems. On the other end was a desire to push for larger, creative improvements aimed at innovating beyond current confines. In addition, we also encountered an innate tension in attempting to weigh the often-divergent perspectives of various stakeholders within the contraceptive clinical trial ecosystem. Because we were developing recommendations for global use, balancing these different perspectives was even more challenging considering the wide range of contexts in which researchers conduct clinical trials, including within different healthcare systems, countries, and resourced settings around the world. We discuss these perspectives in more detail and the implications for engagement with regulatory authorities in Appendix B.

### 4.3. Next steps

To achieve the full benefit of these recommendations, additional dissemination will be necessary. This publication is only the beginning of efforts needed to make these recommendations actual standard practice in contraceptive development. An important next step is to develop tools for stakeholders, such as checklists and guidelines to make adopting these recommendations more straightforward without imposing any unnecessary burden on the development of new contraceptives. It will also be valuable to tailor these resources to the types of research being conducted (e.g., phase I vs. phase III, or clinical vs. social-behavioral research) and to target audiences (e.g., researchers, funders, providers, regulators). We will undertake a concerted and multipronged effort to bring awareness to and promote use of these recommendations across these audiences, including dissemination via presentations, workshops, and discussions at professional meetings, events, and conferences, as well as outreach with traditional scientific media, social media dissemination, participation in virtual meetings and webinars, outreach using author networks, meetings with funders and regulatory authorities, and the creation of an online resource collection with these recommendations and the simplified checklists and guidance mentioned above.

In these next steps, one important limitation of our approach we must acknowledge is the lack of direct input from clinical trial participants in the consensus-building process. Although we aimed to include their perspectives, as understood by our expert group, this does not replace explicit contributions from participants and their communities. To ensure the utility and relevance of the recommendations around future research, this work will need to take a balanced and inclusive approach, including using participatory research methodologies.

In conclusion, we call on stakeholders in the contraceptive clinical trial ecosystem to view these recommendations as new best practices, developed and endorsed by a wide range of experts in contraceptive development and related disciplines. We encourage those involved in the design of clinical trials to begin to incorporate these recommendations into their work as soon as possible.

We also note contraceptives are not the only drug, device, or biologic product that can influence the menstrual cycle, yet such data are not routinely collected in clinical trials or during standard toxicology and pharmacodynamics studies like other organ functions and vital signs. The lack of these data was recently highlighted with the introduction of COVID-19 vaccinations when vaccinated people who could or did menstruate experienced unanticipated bleeding changes [68–72]. We hope our work can be a guide and an invitation to develop recommendations for if, when, and how to measure changes to the menstrual cycle in more types of clinical trials and related research.

Broadly, our consensus recommendations can enable more standardized and patient-centered measurement and analysis of CIMC data in the near term and continued improvement in the future, which can result in more accurate information in contraceptive product labeling on data that matter more to users. Ultimately, such efforts can allow for greater comparability across trials and better data synthesis in systematic reviews and meta-analyses that can inform clinical guidance. In turn, these improvements can enable providers to offer better counseling and users to make more informed decisions about their choice of method, which can improve the experiences of users and better meet the contraceptive needs of people and couples.

## Supplementary Material

2

1

## Figures and Tables

**Table 1:** Characteristics of global experts in topical working groups who developed the recommendations on measuring and analyzing contraceptive-induced menstrual change (CIMC) data in contraceptive clinical trials, 2023 (*N* = 44)

Characteristic	Experts *n* (%)
Gender^a^	
Woman	32 (73)
Man	12 (27)
Age	
Under 40	8 (18)
40–59	21 (50)
60 or over	14 (32)
Area(s) of Training^b^	
Research (PhD, ScD)	25 (57)
Obstetrics and gynecology	16 (39)
Public health practice (DrPh, MPH)	2 (5)
Other	1 (2)
Primary Area(s) of Expertise^b^	
Contraceptive clinical trials/development	27 (61)
Social-behavioral research	17 (39)
Menstrual cycle/menstrual health research	16 (36)
Other contraceptive research	16 (36)
Contraceptive provider/prescriber	12 (27)
Analysis methodologies	7 (16)
Patient-reported outcome measures	5 (11)
Regulatory approval	5 (11)
Years of experience	
6–10 years	7 (10)
11–15 years	6 (14)
16–20 years	11 (25)
More than 20 years	20 (45)
Primary affiliation	
Academic institution	15 (34)
Non-governmental organization	15 (34)
Funder agency/organization	7 (16)
Consultant	3 (7)
Pharmaceutical industry	3 (7)
Total	44

a Self-identified gender as “woman”, “man”, “nonbinary or gender diverse”, or “self-described” in response to the question “With which gender do you identify most?”

b Experts could select multiple, so the sum is greater than 44 (100%).

**Table 2:** Consensus recommendations on measuring and analyzing contraceptive-induced menstrual change (CIMC) data in contraceptive clinical trials from global experts in topical working groups, with shading indicating priorities, 2023 [26–54]

Section 1: Standardization	
**1.1.**	As much as possible, research on CIMCs should aim to: **a.** Simplify terminology and/or align terminology with established standards to ease and improve global communication and collaboration around CIMCs in clinical trials among various stakeholders.^i^ **b.** Collect data and describe CIMCs in a standardized way to allow comparisons across studies and products; researchers may also include assessments of participants’ perceptions of and experience with CIMCs. **c.** Use standardized simple, patient-centered terminology when reporting CIMC data. This terminology should be based on evidence and not be overly prescriptive. In addition, researchers and analysts should include previous analysis approaches and terminology when necessary to permit comparisons to data from previous studies of existing methods.

**1.2.**	Researchers should ideally consider how any new instruments^ii^ used to collect CIMC data relate to commonly used questions in previous trials of already approved contraceptive methods—even if those questions were not part of a validated instrument—to allow comparability of data between current contraceptive methods and new methods for providers, users, and researchers, including for future systematic reviews. *Also see recommendations 2.1 to 2.3.*
***Additional research will be needed for alignment with recommendations 1.1 and 1.2, as well as 2.1 to 2.3 below***.	

**1.3.**	Presently—and until such research is complete—researchers should use the following standardized definitions for spotting and bleeding: **a. Spotting**: Blood and other uterocervical fluid for which the person uses no menstrual products.^iii^ **b. Bleeding**: Any amount of blood and other uterocervical fluid greater than spotting requiring menstrual product use. Researchers should continue to study how diverse populations understand and differentiate spotting and light bleeding to generate more standardized language for measurement across trial populations. *Also see recommendation 2.3b*. *See recommendation 5.2 for additional definitions*.

**1.4.**	While it is important to keep CIMC measures consistent for comparability across trials, there should be some flexibility for local research teams to translate, adapt, and add to these tools based on the local culture and context.
**Section 2: Instruments for measuring CIMCs**	
**2.1.**	Researchers will need to develop new, standard instruments to measure CIMCs in trials because no validated instrument currently exists to address CIMC constructs of interest and for use in the context of contraceptive clinical trials [26]. Instruments for both (a) the menstrual changes themselves (e.g., changes in bleeding frequency, duration, volume; change in blood; changes in pain) and (b) the impact, acceptability^iv^, and perceptions of those menstrual changes will be needed. Development of new, standard instruments can include: (a) generating and validating new items, and/or (b) modifying items from related, existing instruments used to measure changes to the menstrual cycle (e.g., instruments developed within the context of examining menstrual & gynecological disorders & symptoms).

***Because additional research will be needed to develop and validate instruments in alignment with recommendation 2.1, recommendations 4.1 to 4.13 describe data collection approaches recommended for trials conducted prior to this additional research and recommendations 5.1 to 5.13 describe related data analysis approaches***.	
*Recommendations 2.2 and 2.3 provide additional details on instrument development and validation. Also see recommendation 1.3*.	

**2.2.**	When developing instruments to measure CIMCs in trials, it is critical that researchers: **a.** Follow current recommendations from regulatory agencies and professional societies and consortia, such as the United States Food and Drug Administration’s guidance on patient-reported outcome^v^ use in trials [15] and the Patient-Reported Outcomes Tools: Engaging Users and Stakeholders Consortium’s recommendations on measuring patient-reported outcomes effectively.^vi^ **b.** Use simple, evidence-based patient-centered terminology both within instrument items, prioritizing the perspective of the contraceptive user and simplicity over the convenience of the researcher or imposing definitions or characterizations that may not be sufficiently objective to translate across contexts.^vii^ **c.** Ensure instruments are accessible to those with differing literacy levels and people with disabilities, including consideration of pictorial instruments and adherence to current United States Government plain language guidelines [44] and Web Content Accessibility Guidelines [45] for digital content. **d.** Ensure instruments adhere to data privacy requirements, legal protections, and ethical considerations to protect data and prevent use of data by any entity outside of those described in the trial informed consent forms.

**2.3.**	When developing instruments to measure CIMCs in trials, ideally: **a.** For participant-entered diary instruments, researchers should establish evidence^viii^ on recall to determine the window of retrospective data entry permitted for each type of CIMC measured; such evidence may build upon previous research around heavy menstrual bleeding. **b.** Researchers should establish the transferability^ix^ of instruments, including following recommendations such as the Professional Society for Health Economics and Outcomes Research principles on good practice for translation and cultural adaptation of patient-reported outcomes [46,47] because instruments are needed in different languages and sociocultural contexts, including for multisite trials. When feasible, this should include simultaneous development and validation of instruments in multiple contexts. (*Also see recommendation 1.3*.) **c.** Researchers should consider time and cost requirements to implement instruments, so they do not unduly hinder use in contexts with fewer resources. **d.** For mixed mode instruments (e.g., paper and electronic instruments, or interactive voice response and short message service/chat instruments), researchers should establish equivalence^x^ between modes (e.g., between paper and electronic versions of an instrument), including following recommendations such as the Professional Society for Health Economics and Outcomes Research good research practices on use of mixed mode patient-reported outcomes [48]. **e.** For instruments requiring data entry by participants on electronic hand-held devices (i.e., phones or tablets), researchers should establish equivalence^x^ for devices provided to participants by the trial as well as for devices already used/owned by participants (i.e., ‘bring your own’ devices) so either can be used by trials without the potential of introducing differential bias between trials providing devices and trials with ‘bring your own’ devices or within trials where some participants are provided devices and some are ‘bring your own’ devices [49]. **f.** Researchers should consider if additional instrument items or modules are needed for participants from populations who may have different CIMC outcomes and experiences (e.g., those with menstrual & gynecological disorders & symptoms).
**Section 3: Trial design, protocol development, participant recruitment**	
**3.1.**	Early phase studies should consider evaluation of drug-drug interactions—including pharmacokinetic or pharmacodynamic interactions that may impact CIMCs—for commonly used medications that may influence CIMCs. These medications can include over the counter, supplements, or even certain foods.

**3.2.**	Every effort should be made to evaluate a range of body mass index^xi^ categories in pharmacokinetic studies, as body mass index heterogeneity can have major implications for pharmacokinetic and/or CIMCs.
**3.3.**	Related to intrinsic or extrinsic factors that may impact CIMCs: **a.** Researchers should consider the most relevant intrinsic or extrinsic factors and design enrollment for Phase III and IV trials to include adequate numbers of participants with these factors. The choice of relevant factors will be informed by trial context, trial population, product characteristics, and/or trial design. **b.** Information on intrinsic and extrinsic factors should be collected during the trial, as well as at enrollment. **c.** When the primary trial outcome is pregnancy prevention, researchers should consider the impact of the selected intrinsic and extrinsic factors on risk of pregnancy.^xii^

**3.4.**	Researchers should establish and standardize what data are needed at enrollment on menstrual cycle history (when not using hormonal or intrauterine contraception) and other sexual and reproductive health history based on the CIMC data collected during the trial in order to permit comparisons between baseline and treatment outcomes. Additional baseline data on sociodemographics and other relevant background data may also be needed for stratified analyses to examine relevant intrinsic and extrinsic factors that may impact CIMCs.
**3.5.**	When feasible, in Phase II and III trials, measurement of CIMCs and related outcomes should include input from local researchers, and whenever possible, also be under the advisement of local community advisory boards and community advocates. This input should incorporate input from a variety of stakeholders, including partners and families.
**3.6.**	In Phase III and IV studies, diverse trial sites should be used to more fully represent differences in CIMC experiences and sociocultural differences in menstruation perceptions/knowledge across populations of future users.^xiii^

**3.7.**	Phase IV studies should be designed to build upon Phase III findings, to collect data on additional CIMC-related factors that may influence tolerability and method continuation, and to include adequate numbers of participants with these factors. The factors of interest may differ by trial context, product characteristics, and/or trial design.

**3.8.**	All health care providers who will be involved in contraceptive clinical trials should be trained in comprehensive counseling methods, including providing information about different CIMCs and management strategies for CIMCs that are culturally and contextually appropriate and referring to appropriate services when indicated.
**Section 4: Data collection**	
***Additional research will be needed to align with recommendations 2.1 to 2.3. For trials conducted prior to this additional research, recommended data collection approaches are presented in recommendations 4.1 to 4.13 and recommended analysis approaches for these data are presented in recommendations 5.1 to 5.13***.	

**4.1.**	Presently, researchers should include two types of assessments for primary outcomes of CIMCs: **a.** Bleeding and spotting days (*see* *recommendation 1.3*), and **b.** Frequency, duration, and volume of bleeding episodes (*see* *recommendation 5.2*).

**4.2.**	Presently, researchers may also collect data on additional patient-reported outcomes such as cramping, pain, changes in blood and other uterocervical fluid, as well as other symptoms that have been shown to influence patient experiences with and perceptions of contraceptive methods. *See recommendations 4.11 to 4.13 for details*. More research is needed to create validated instruments for these types of additional outcomes across trial contexts. *See recommendations 2.1 to 2.3 for details on instrument development and validation recommendations*.

**4.3.**	Presently, researchers should collect bleeding and spotting data in trial phases as follows: **a.** In Phase I and IIa trials, researchers may choose to capture bleeding and spotting assessments as an exploratory outcome to make early decisions about product viability. **b.** In Phases IIb and III, researchers should collect bleeding and spotting data. **c.** In Phase IV, bleeding and spotting assessments may be required to assess additional use/benefits of the contraceptive (e.g., for special populations, health conditions, comparisons with other contraceptives).
**4.4.**	Presently, researchers should collect data on frequency, duration, and volume of bleedine episodes in trial phases as follows: **a.** In phases IIb and III, these assessments are recommended. **b.** In phase IV, these assessments may be required for additional use/benefits of the contraceptive (e.g., for special populations, health conditions, comparisons with other contraceptives).

**4.5.**	Presently, researchers should collect some bleeding data for the entire duration of the method, as practical, including for methods with longer durations (e.g., 3-5 years).

**4.6.**	Presently, researchers should use validated measures for an objective measure of bleeding volume in trials when bleeding volume is an important outcome for label purposes (e.g., as an indication for heavy menstrual bleeding).

**4.7.**	Presently, researchers should design bleeding data capture instruments to register information daily when possible and, if not, at least each time when bleeding or spotting occurs. However, researchers should study alternatives to daily collection of data for methods that have long durations (e.g., 3-5 years). *See recommendation 2.3a on recall for new instruments*.
**4.8.**	Presently, when using electronic diaries, the diaries should be designed to allow a window of time for users to retroactively record bleeding occurrences (e.g., within 48 hours); however, more research needs to be conducted to find the optimal window of time. *See recommendation 2.3a on recall for new instruments*.

**4.9.**	Presently, researchers should consider the culture and context of their trial setting to determine whether paper or electronic diaries will be more acceptable and/or feasible and assess the strengths and limitations of each method of data collection. *See recommendation 2.3d on mixed modes for new instruments*.

**4.10.**	Presently, researchers using electronic diaries should attempt to incorporate data capture on a participant’s own phone to avoid the burden of using additional devices where possible, despite software standardizing challenges. *See recommendation 2.3e on are ‘bring your own’ devices for new instruments*.

**4.11.**	Presently, trials should measure acceptability and quality of life related to CIMCs to assess the impacts of CIMCs on users and inform contraceptive counseling and user choices. This can be done in the following ways: **a.** In Phase I, II, and III, researchers should measure participants’ self-perceived changes in bleeding and other CIMCs (e.g., whether participants self-define changes as significant). This should be measured over time for Phase II and III. **b.** In Phase II and III, researchers should measure the physical and psychosocial impacts of CIMCs on quality of life, including burdens and benefits. The aspects that are measured should be based on local priorities and informed by local researchers and advisors, and may include impacts on work, sexual well-being, mental burden, anxiety and stress, quality of relationships, social well-being, and financial well-being (including the burden of menstrual health management). **c.** In Phase II and III, researchers should measure changes in acceptability related to CIMCs over time.

**4.12.**	Presently, in Phase III, and when possible in Phase II, trials should include measures to contextualize and understand the CIMC acceptability and quality of life impacts outlined in recommendation 4.11. This can be done in the following ways: **a.** Trials should collect sufficient demographic data, including about race and ethnicity, socioeconomic status, age, disability, sexual orientation, gender identity, location (e.g., urban/rural), and other social factors. **b.** Trials should collect data about affordability and access to menstrual health information, menstrual products/materials, and water and facilities for changing, washing, and disposing of menstrual products. **c.** Trials should collect data about the ethicality and cultural appropriateness of CIMCs (e.g., shame or stigma surrounding CIMCs) in the trial context, along with individuals’ beliefs about CIMCs (e.g., the relationship between CIMCs and long-term health outcomes like infertility or cancer). **d.** Researchers should measure self-efficacy related to managing CIMCs, that is, an individual’s confidence in their capacity to manage the CIMCs experienced.
**4.13.**	Presently, in all phases of trials, acceptability and quality of life impacts of CIMCs should be measured using both quantitative measures and qualitative methods whenever possible and advantageous. Qualitative methods will vary based on context and type of trial but may include open-ended survey questions, in-depth interviews, or ranking exercises. Research teams should include members experienced in qualitative measurement and analysis whenever these methods are being used.
**Section 5: Data analysis**	
***Additional research will be needed to align with recommendations 1.1, 1.2 and 2.1 to 2.3, which will include establishing how new instrument(s) will be analyzed. For trials conducted prior to this additional research, recommended data analysis approaches are presented in recommendations 5.1 to 5.13 for data collected per recommendations 4.1 to 4.13***.	
**5.1.**	Presently, to align with a patient-centered approach, data in the recommendations below should be reported for each 30-day **reference period**. To permit comparison to existing methods, data should also be reported for each 90-day **reference period**, as well as other reference period length if relevant for the method.^xiv^

**5.2.**	Presently, researchers should use the following definitions during analysis for bleeding episode and for duration, frequency, and volume of bleeding episodes: **a. Bleeding episode**: one or more consecutive days with bleeding and/or spotting, bordered by 2 full days without any bleeding or spotting. **b. Duration**: A bleeding episode can be classified into prolonged or not prolonged. • *Prolonged Duration*: Bleeding/spotting episode lasting more than 7 days. **c. Volume:** Volume should be classified according to the participant’s perception. Volume should be described as “lighter,” “usual,” or “heavier” as compared to the individual’s typical volume when not using contraception. **d. Frequency**:^xv^ • *Absence of bleeding/spotting*: no bleeding or spotting during the reference period. • *Infrequent bleeding*: 2 or fewer bleeding/spotting episodes durine a 90-day reference period. • *Frequent bleeding*: > 4 bleeding/spotting episodes during a 90-day reference period. • *Scheduled bleeding*: a bleeding/spotting episode starting during the expected interval and lasting no more than 7 days for a method expected to have a predictable bleeding pattern.^xvi^ • *Unscheduled bleeding*: a bleeding/spotting episode occurring at any time prior to the expected interval, even if it continues into the next expected interval for a method expected to have a predictable bleeding pattern.^xvi^
**5.3.**	Presently, analysts should report the following **variables** on number of bleeding and spotting days during the reference period: **a.** Total number of bleeding or spotting days, **b.** Number of bleeding days, **c.** Number of spotting days, **d.** Number of scheduled and unscheduled bleeding or spotting days, if relevant for the method as described in recommendation 5.2d.

**5.4.**	Presently, analysts should report the following **descriptive statistics** on number of bleeding and spotting days during the reference period: **a.** For Phase I and IIa trials, as exploratory summary statistics: • Mean, median, interquartile range for: ○ Bleeding or spotting days, ○ Bleeding days, ○ Spotting days, ○ Instances of consecutive days with no bleeding if daily bleeding data were collected, and ○ Scheduled and unscheduled bleeding or spotting days, if relevant for the method as described in recommendation 5.2d. • Percent of participants with no bleeding or spotting. **b.** For Phase IIb, III and IV trials: • Mean, median, range, interquartile range, • An estimate of precision (e.g., standard error of the mean or confidence interval) • These data should be presented separately for total, unscheduled, and scheduled days if relevant for the method as described in recommendation 5.2d. However, analysts are advised to consider whether the sample size and presumed distribution of the data warrants the use of standard statistical methods.

**5.5.**	Presently, analysts should report the following **variables** on bleeding frequency during the reference period. **a.** To align with a patient-centered approach (*see recommendation 5.1*): • Whether bleeding episodes occur (1) More than once per month, (2) Once per month, or (3) There is a month without bleeding • Whether there were no bleeding episodes for three or more months in a row, if relevant for the method **b.** To permit comparison to data from existing methods using a 90-day reference period: • No bleeding or spotting • Infrequent bleeding^xv^ • Frequent bleeding^xv^ • Scheduled and unscheduled bleeding or spotting, if relevant for the method as described in recommendation 5.2d
**5.6.**	Presently, analysts should report the following **variables** on bleeding duration during the reference period. **a.** To align with a patient-centered approach (*see recommendation 5.1*): Mean, median, interquartile range for number of bleeding days per bleeding episode. **b.** To permit comparison to data from existing methods using a 90-day reference period: The number of participants with prolonged bleeding. **b.** Bleeding duration variables should be reported separately for scheduled and unscheduled bleeding episodes, if relevant for the method as described in recommendation 5.2d.
**5.7.**	Presently, analysts should report the following **variables** on bleeding volume during the reference period. **a.** To align with a patient-centered approach (*see recommendation 5.1*): An assessment by the participant per recommendation 5.2c **b.** If indirect quantitative or semi-quantitative measurement of volume of bleeding is needed for the trial, analysis will depend on how data are collected (i.e., alkaline hematin assay. Pictorial Blood Assessment Charts). *See recommendation 4.6*. **c.** Bleeding volume variables should be reported separately for scheduled and unscheduled bleeding episodes, if relevant for the method as described in recommendation 5.2d.
**5.8.**	Presently analysts should report the following **descriptive statistics** on bleeding frequency and bleeding duration during the reference period: **a.** For Phase I and IIa trials: same as Phase IIb, III, and IV trials when the trial is longer than 90 days. **b.** For Phase IIb, III, and IV trials: Absolute and relative frequencies of the variables for frequency and duration of bleeding episodes
**5.9.**	Presently, analysts should report the following **inferential statistics** on number of bleeding and spotting days, as well as frequency, duration and volume of bleeding, during the reference period: **a.** For Phase I and IIa trials: do not include any formal hypothesis testing, but exploratory analysis could be used to exclude contraceptives with undesirable CIMCs. **b.** For Phase IIb, III and IV trials: Use hypothesis testing for studies with comparator/control and use an analysis method that accounts for repeated measures on subjects, such as generalized estimating equations.

**5.10.**	Presently, for Phase IIb, III, and IV trials, analysts should report **sensitivity analysis** to evaluate the effect of some variables of interest on bleeding and spotting (e.g., age, body mass index, smoking status). If feasible and informative, sensitivity analyses can be done for Phase I and IIa trials.

**5.11.**	Presently, analysts should report the following about **missing data**^xvii^ on the number of bleeding and spotting days, as well as frequency, duration and volume of bleeding, during the reference period: **a.** For Phase I and IIa trials: The number of discontinuations and the reasons for discontinuation **b.** For Phase IIb, III and IV trials: The number of discontinuations and the reasons for discontinuation; and the potential impact of informative drop-out when describing CIMCs over time if a meaningful percentage of trial participants discontinue due to CIMCs^xviii^

**5.12.**	Presently, analysts should include **data visualizations**, such as heatmaps and boxplots, for data from all participants for all trials.
**5.13.**	Data on CIMC acceptability should be analyzed as dictated by the instrument used for measurement. Qualitative data should be analyzed via standard qualitative methods appropriate for the qualitative approach.

**5.14.**	Following analysis, researchers/analysts should: (a) deposit deidentified data in an established open data repository, as soon as possible; (b) deposit deidentified CIMC data from assays in an established open data repository; and (c) aim to make instruments openly available for broad use, ideally at no cost. These steps should be done whenever possible, such that the community can benefit from shared knowledge and minimized duplication of resources.^xix^
**Section 6: Areas for exploratory research**	
**6.1.**	When developing non-patient-reported outcome instruments to measure CIMCs in trials to advance research on biospecimen collection and assays: Researchers should establish a target assay profile^xx^ for instruments used by trial investigators and/or participants for biospecimen collection and assays. Example ideal characteristic criteria could be affordability, acceptability, ease of storage without cold chain requirement, low burden on participants and/or trial investigators (e.g., non-invasive, ease of collection/assay), speed of results, and ease of interpretability. Urine pregnancy tests can be used as a model for biospecimen collection and assays.
**6.2.**	To advance a future research agenda for measurement of CIMCs in trials, researchers should explore avenues for developing new or improved assays and identifying biomarkers^xxi^ or other approaches such as predictive modeling for CIMCs. However, researchers should always prioritize protecting participant privacy and data security when considering the use of new methodology, especially when using Artificial Intelligence or Big Data approaches [50–54].

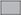
 85% of experts deemed to be a priority based on their urgency and the potential influence of their implementation

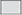
 75% of experts deemed to be a priority based on their urgency and the potential influence of their implementation

i. These standards can include the International Federation of Gynecology and Obstetrics System 1 for nomenclature of normal and abnormal uterine bleeding [55], the Global CIMC Task Force’s definition of CIMCs [1]. recommendations on bleeding data analyses in contraceptive studies [12], and other terminology harmonization efforts, especially those using methodology to establish consensus and priorities (e.g., Delphi method, Child Health and Nutrition Research Initiative approach).

ii. Definition of instrument: An approach or method to measure or assess the outcome of interest, including those that are: (a) participant/patient-reported, (b) researcher/clinician-reported, (c) observer-reported; (d) biomarker-based, or (e) assay-based, as well as both ‘objective’ or ‘subjective’ measurements that encompass both direct observations and/or perceptions (Adapted from [56])

iii. In some populations, the availability of menstrual products may be limited and thus reduce their use. Other populations may readily access menstrual products and use them differently than other populations would. When a trial is performed in such a population, the authors should describe this issue and how they choose to define spotting and bleeding for their particular population.

iv. Acceptability [of CIMCs] refers to a multidimensional concept wherein a user considers the CIMCs they experience to be suitable based on their individual circumstances. When considering whether a CIMC is acceptable, a user may evaluate their satisfaction and comfort with the CIMC, feelings regarding the burden/impact related to the CIMC, and the extent to which the CIMC fits within their value system [4,57]. By characterizing a CIMC as acceptable, a user effectively considers the CIMC to be suitable based on their individual anticipated, perceived, or experiential responses [7]. Acceptability will be influenced by a user’s own unique attitudes and context. It is shaped by individual and community-level norms, preferences, and practices regarding menstrual health management and self-efficacy to manage the CIMC.

v. All data on CIMCs in trials are reported by the participant (i.e., it is a patient reported outcome unless the trial is using an indirect quantitative and semi-quantitative approach like an alkaline hematin assay to determine menstrual blood loss from used menstrual products. See 4.6, 5.7b, 6.1, and 6.2 for recommendations on these types of instruments.

vi. Patient-Reported Outcomes Tools: Engaging Users and Stakeholders Consortium recommendations on measuring patient-reported outcomes effectively with standards from the International Society for Quality of Life Research [58].

vii. If supported by evidence, an example of this could be asking participants about use/nonuse of menstrual products, instead of asking participants to use and understand the term ‘spotting’, which may not be relevant in all contexts.

viii. “Establishing evidence” can include reviewing existing research and/or conducting new research.

ix. Transferability is the degree to which an instrument can be transferred between linguistic and sociocultural groups.

x. Measurement equivalence is a function of the comparability of the psychometric properties of the data obtained via the original and adapted administration mode… to demonstrate that the change did not introduce response bias and/or negatively affect the measure’s psychometric properties” [48].

xi. There are many critiques of body mass index as an indicator and important concerns about its history and use [59–62]; however, we also note current regulatory use of body mass index, including in US Food and Drug Administration requirements for hormonal contraceptive clinical trials (i.e,, “The effectiveness of some contraceptives may be reduced with increasing body weight. Sponsors should not place restrictions on body mass index (BMI) for trial enrollment. The trial population should include obese women (i.e., defined as BMI of at least 30 kg/m^2^), and the analysis plan should include a prespecified subgroup efficacy analysis in this population, Insufficient data in the obese population may result in a limitation of use for this population in labeling. During the trial design phase, sponsors should discuss with the division the adequacy of the number of cycles of drug exposure that will be derived from obese subjects.”; and “For the overall trial population, as well as for those younger than or equal to 35 years old at study enrollment and those older than 35 years old, sponsors should perform subgroup analyses [of efficacy] based on BMI at study enrollment….)” [63].

xii. Researchers may consider the factor of age as an example for how these relevant intrinsic and extrinsic factors that may impact risk of pregnancy could be addressed in trials (i.e., US Food and Drug Administration requirements for hormonal contraceptive clinical trials: “Trials should include subjects from all premenopausal age groups who are likely to use the drug product, including postmenarchal adolescents.”; “The primary efficacy results should be calculated using the trial population of women younger than or equal to 35 years old at study enrollment because the likelihood of pregnancy decreases with advancing age. Include additional efficacy analyses for the overall trial population and a subgroup analysis for those older than 35 years old.” and “Enrollment of subjects older than 35 years old is recommended for safety determinations. The number of subjects older than 35 years old who should be enrolled in the trial or trials will depend on the existing experience with the drug product ingredients and should be discussed with the division [of Bone, Reproductive, and Urologic Products in the Center for Drug Evaluation and Research at the Food and Drug Administration]” [63].

xiii. This site diversity may go beyond Stringent Regulatory Authorities minimums and should include recruiting and enrolling participants within and between trial sites from different racial and ethnic groups, socioeconomic statuses, age categories, disability status, sexual orientation, and gender identity, as these all may influence CIMC experiences and perceptions of menstruation.

xiv. For example, 28 days for a combine oral contraceptive with 24/4 regimen (i.e., 24 days of pills with the APIs and 4 days of placebo pills) or other single cycle regimens (e.g., 21/7); or 84 days for an extended cycle combined oral contraceptive [12].

xv. We have not included a “normal” frequency definition. Earlier versions of this recommendation included a definition for “approximately monthly bleeding” as “3 or 4 bleeding/spotting episodes during the 90-day reference period”, similar to that proposed by Creinin et al. [12]. Without clarification on the duration between these episodes, however, some experts raised the concern that a bleeding pattern could fit this definition but not actually be “monthly” or “normal” (e.g., 3 or 4 episodes in the first 30 days and then none in the latter 60 days). See 5.5a for an alternative, patience-centered frequency definition. Further work towards consensus on an appropriate definition should be part of future updates to these recommendations.

xvi. Predictable bleeding pattern: an expected predictable pattern is based on the product design as determined by the regimen (e.g., combined hormonal methods [oral, transdermal, vaginal, or injectable] and non-hormonal methods such as intrauterine devices or non-hormonal vaginal products). Unpredictable bleeding pattern: an expected unpredictable pattern based on the product design (e.g., progestin only methods including implants, intrauterine devices, injectables, pills, patches, and rings) [12].

xvii. Data may be missing due to early method discontinuation, trial discontinuation, or loss to follow up.

**xviii.:** This is to account for the distribution of CIMC outcomes towards the end of the trial being disproportionately represented by subjects who did not experience those outcomes or experienced less impactful outcomes. Approaches could include (a) reporting CIMC outcomes separately among all enrolled participants (where the denominator is the number of subjects still in follow-up at a given time) and among participants who completed the trial (where the denominator is uniform over time), where differences in patterns would suggest that informative drop-out was biasing the results; or (b) explicitly modeling the (potentially informative) drop-out mechanism using an appropriate methodology, such as the one developed and performed in the context of depot medroxyprogesterone acetate-induced amenorrhea [64,65] using weighted generalized estimation equations for population-level analyses [66].

xix. There are notable concerns about the extent to which data can be truly deidentified, especially as larger amounts of open data continue to become available [67–69].

xx. This can be analogous to a drug/device target product profile with minimum acceptable result and ideal results (e.g., [70]).

xxi. Developing assays and biomarkers for trials should be done in consultation with regulatory agencies.
