# Supplementary material for: Consensus recommendations for measuring the impact of contraception on the menstrual cycle in contraceptive clinical trials
Source: Contraception. Author manuscript; Available in PMC 2026 May 26. (PMC13210849; doi:10.1016/j.contraception.2025.110829)
Supplement: 2 [file NIHMS2062258-supplement-2.pdf]

Appendix B

This Appendix provides additional details on areas of contention we encountered during our consensus-building process and suggestions for implementing recommendations based on these conversations, including the considerations for, and engagement with, regulatory authorities.

On one hand, recommendations needed to be specific and technical enough to provide actionable guidance to trial investigators without being too burdensome to enact within the many constraints and competing demands of conducting a clinical trial. From this researcher perspective, there can be an appeal of smaller, incremental improvements to current practices that could be simple to implement with often-limited resources. This gradual approach could also be appealing from a sponsor and funder standpoint because it may not require increased trial budgets and may be more likely aligned with current and familiar regulatory strategies. For some experts, these types of modest changes within current norms and systems were of interest for very practical, realistic reasons founded in their many years of experience. Indeed, complex and/or numerous novel recommendations that may demand additional time and funding could result in delaying upcoming trials, as well as fewer, longer trials in the future. In addition, many experts were concerned our recommendations would be interpreted by funders or regulators as expert-endorsed mandates that should be required for every trial. Altogether, the broader result of these types of additional burdens and constraints could be fewer new methods being developed, which could ultimately mean the contraceptive needs of some people and couples may not be met. To address the concerns our recommendations could hinder contraceptive research and development, we specify they should not be considered required for contraceptive trials to be funded, conducted, or reviewed by regulatory authorities. Rather, stakeholders should consider these as recommended approaches to achieving standardization and comparability across trials and to guiding future research for improvement, all with emphasis on identified priorities.

On the other hand, this convening of around 50 interdisciplinary experts from multiple global regions in a consensus-building process was a unique opportunity to push the contraceptive clinical trial ecosystem to consider innovative CIMC data collection and analysis approaches that could be more informative to providers in counseling on new contraceptives and more relevant to future users. Many experts endorsed this perspective, which is also aligned with current patient-focused drug development initiatives, other efforts at increasing patient engagement in clinical

trials and regulatory decisions, and wider patient-centered outcome research [1–6]. As described in our recommendations, research will be needed to align CIMC measurement with best practices for patient-reported outcomes. Because this approach is novel for contraceptive trials—although increasingly common in other therapeutic areas—it will require close adherence to regulatory guidelines and engagement with regulators.

References

[1] US Food and Drug Administration. Patient-Focused Drug Development: Collecting Comprehensive and Representative Input: Guidance for Industry, Food and Drug Administration Staff, and Other Stakeholders 2020. https://www.fda.gov/regulatory-information/search-fda-guidance-documents/patient-focused-drug-development-collecting-comprehensive-and-representative-input (accessed July 31, 2024).

[2] US Food and Drug Administration. Patient-Focused Drug Development: Methods to Identify What Is Important to Patients: Guidance for Industry, Food and Drug Administration Staff, and Other Stakeholders 2022. https://www.fda.gov/regulatory-information/search-fda-guidance-documents/patient-focused-drug-development-methods-identify-what-important-patients (accessed July 31, 2024).

[3] US Food and Drug Administration. Patient-Focused Drug Development: Selecting, Developing, or Modifying Fit-for-Purpose Clinical Outcome Assessments: Draft Guidance for Industry, Food and Drug Administration Staff, and Other Stakeholders 2022. https://www.fda.gov/regulatory-information/search-fda-guidance-documents/patient-focused-drug-development-selecting-developing-or-modifying-fit-purpose-clinical-outcome (accessed July 31, 2024).

[4] US Food and Drug Administration. Patient-Focused Drug Development: Incorporating Clinical Outcome Assessments Into Endpoints for Regulatory Decision-Making 2023. https://www.fda.gov/regulatory-information/search-fda-guidance-documents/patient-focused-drug-development-incorporating-clinical-outcome-assessments-endpoints-regulatory (accessed July 31, 2024).

[5] Gnanasakthy A, Qin S, Norcross L. FDA Guidance on Selecting, Developing, or Modifying Fit-for-Purpose Clinical Outcome Assessments: Old Wine in a New Bottle? The Patient - Patient-Centered Outcomes Research 2023;16:3–5. https://doi.org/10.1007/s40271-022-00607-6.

[6] The Council for International Organizations of Medical Sciences. Patient involvement in the development, regulation and safe use of medicines. Geneva: 2022. https://doi.org/10.56759/iiew8982.
