# Supplementary material for: Consensus recommendations for measuring the impact of contraception on the menstrual cycle in contraceptive clinical trials
Source: Contraception. Author manuscript; Available in PMC 2026 May 26. (PMC13210849; doi:10.1016/j.contraception.2025.110829)
Supplement: 1 [file NIHMS2062258-supplement-1.pdf]

Appendix A

Appendix Figure A.1. Interim consensus-building outcomes for recommendations on CIMC measurement in contraceptive clinical trials

Topics considered for the scope of recommendations	Agreement for inclusion	Translated into	Experts [1]
Eligibility and enrollment requirements	92%	Working Group 1: Eligibility<br>•Eligibility & enrollment requirements<br>•Confounders	Sharon Achilles, Anne Burke [3], Enrico Colli, Hilary Critchley, Laneta Dorflinger, Chukwuemeka Nwachukwu, Kevin Peine, Lisa Soule
Data collection on confounders and other variables	95%		
Frequency of CIMC data collection	100%	Working Group 2. Data<br>•Type, frequency, and format/mode of CIMC data collected	Diana Blithe, Vivan Brache, Amanda Cordova-Gomez, Cássia Juliato, Nora Miller [3], Kavita Nanda, Régine Sitruck-Ware, Carolina Sales Vieira
CIMC instrument format and mode	95%		
Type of CIMC data collected during clinical trials	100%		
Measurement of acceptability and impact on daily life	100%	Working Group 3. Acceptability<br>•Measurement of acceptability & impact on daily life<br>•Culture & context [2]	Rebecca Callahan, Kate Clancy, Lisa Haddad, Julie Hennegan, Emily Hoppes [3], Simon Kibira, Alex Mickler, Funmilola OlaOlorun, Jenni Smit, Marsden Solomon, Betsy Tolley, Olivia Vandeputte
Culture and context [2]	87%		
Developing new CIMC instruments	85%	Working Group 4. Instruments<br>•Research agenda for developing CIMC instruments	Leigh Allen, Alice Cartwright, Betsy Costenbader, Gustavo Doncel, Amelia Mackenzie [3], Diana Mansour, Kristen Matteson, Jackie Maybin, Chelsea Polis
Measures and analyses standardization	97%	Working Group 5. Analysis<br>•Analysis methodology<br>•Measures & analyses standardization	Luis Bahamondes, Mitchel Creinin, Alison Edelman, Thomas Faustmann, Christoph Gerlinger, Amelia Mackenzie [3], Andres Martinez, Carolina Sales Vieira, Douglas Taylor, Leigh Wynne [3]
Analysis methodology	85%		
Regulatory considerations	85%	Three Cross-Cutting Themes<br>1. Regulatory considerations<br>2. Culture & context [2]<br>3. Incorporating user perspectives & understandings of CIMCs	
Culture and context†	87%		
Improved counseling information [4]	56%		
Guiding frameworks (reproductive justice, life course, equity)	47%	Did not reach consensus during voting	
Trial sample characterization (life stage, fertility intensions, goals/expectations, desires/needs)	50%		
Non-contraceptive use (label information, menstrual choice, impact on wider health)	47%		
Menstrual changes data in other trials beyond contraception	47%		
CIMCs in pre-clinical development [5]	19%		
Expanding the definition of "acceptability" of contraception	N/A	Determined to be out-of-scope during Working Group discussions [5]	
Balancing efficacy and acceptability data to support contraceptive counseling			
Coherence			
Sexual pleasure and well-being			

[1] Ad hoc advisors who provided specific guidance to working groups include Lisa Rarick (regulatory), Greg Kopf (preclinical and early pharmaceutical trials), and Marni Sommer (menstrual health)

[2] Culture & context: both under the prevue of Working Group 3 and a cross-cutting theme for all working groups to consider in recommendations

[3] Working group chair(s)

[4] Revised into "Incorporating User Perspectives and Understandings of CIMCs" and agreed upon as cross-cutting theme

[5] **CIMCs in pre-clinical development**: Debate about if the expert consultation scope should include pre-clinical development research such as use of animal models that menstruate, as well as organoids, physiomimetics, other in vitro organ systems; **Expanding the definition of "acceptability" of contraception**: Discussion around how we generally define acceptability, and if we want to radically expand the definition with a higher bar to push acceptability beyond "minimally acceptable"; **Balancing efficacy and acceptability data to support contraceptive counseling**: Role of expert consultation in advocating for contraceptive clinical trials to emphasize acceptability data in conjunction with efficacy data, specifically in an effort to support clinicians in counseling efforts; **Coherence**: (i.e., the extent to which the participant understands how the intervention works) as an important domain to measure in contraceptive clinical trials; **Sexual pleasure and well-being**: Discussion around the importance of including sex and pleasure and the positive aspects of contraceptive use when measuring acceptability and preferences in clinical trials.

Appendix Table A.1. Consensus agreement and priorities for recommendations on CIMC measurement in contraceptive clinical trials via Questionnaire 3, Day 4 meeting, and the Prioritization Survey

Recommendation	Consensus agreement	Stage reached consensus	Priority agreement
**Section 1**			
1.1	a: 100%<br>b: 92%<br>c: 92%	Questionnaire 3	100%
1.2	100%	Questionnaire 3	67%
1.3	a: 78%<br>b: 85%<br>c: 100%	Prioritization Survey<br>Questionnaire 3<br>Questionnaire 3	78%
1.4	100%	Questionnaire 3	61%
**Section 2**			
2.1	100%	Questionnaire 3	78%
2.2	a: 92%<br>b: 100%<br>c: 100%<br>d: 100%	Questionnaire 3	100%
2.3	a: 88%<br>b: 96%<br>c: 92%<br>d: 95%<br>e: 88%<br>f: 96%	Questionnaire 3	61%
**Section 3**			
3.1	81%	Questionnaire 3	28%
3.2	96%	Questionnaire 3	83%
3.3	a: 89%<br>b: 100%<br>c: 94%	Day 4 meeting<br>Questionniare 2<br>Prioritization Survey	78%
3.4	100%	Questionnaire 3	72%
3.5	88%	Questionnaire 3	56%
3.6	88%	Questionnaire 3	72%
3.7	96%	Questionnaire 3	83%
3.8	77%	Questionnaire 3	72%
**Section 4**			
4.1	92%	Questionnaire 3	94%
4.2	96%	Questionnaire 3	56%
4.3	a: 85%<br>b: 89%<br>c: 89%	Questionnaire 3	78%
4.4	92%	Day 4 meeting	83%
4.5	85%	Questionnaire 3	67%
4.6	94%	Day 4 meeting	78%
4.7	92%	Questionnaire 3	67%
4.8	92%	Questionnaire 3	72%

Recommendation	Consensus agreement	Stage reached consensus	Priority agreement
4.9	100%	Questionnaire 3	78%
4.10	88%	Questionnaire 3	56%
4.11	3.1: 96%<br>a: 96%<br>b: 95%<br>c: 92%	Questionnaire 3<br>Questionnaire 3<br>Day 4 meeting<br>Questionnaire 3	89%
4.12	3.2: 92%<br>a: 92%<br>b: 81%<br>c: 81%<br>d: 85%	Questionnaire 3	61%
4.13	92%	Questionnaire 3	67%
**Section 5**			
5.1	85%	Questionnaire 3	61%
5.2	a: 100%<br>b: 85%<br>c: 85%<br>d: 96%/89% [1]	Questionnaire 3	100%
5.3	a. 92%<br>b. 92%<br>c. 85%<br>d: 85%<br>e: Not reached [2]	Questionnaire 3<br>Questionnaire 3<br>Questionnaire 3<br>Questionnaire 3<br>Prioritization Survey	94%
5.4	a: 83%<br>b: 81%	Prioritization Survey<br>Day 4 meeting	78%
5.5	a: 81-85% [3]<br>b: 81-89% [4]	Questionnaire 3	72%
5.6	a: 77%<br>b: 85% [5]<br>c: 92%	Questionnaire 3	56%
5.7	a: 81%<br>b: 95%<br>c: 92%	Questionnaire 3<br>Day 4 meeting<br>Questionnaire 3	56%
5.8	91%	Day 4 meeting	72%
5.9	88%	Day 4 meeting	56%
5.10	100%	Day 4 meeting	78%
5.11	77%	Questionnaire 3	94%
5.12	88%	Questionnaire 3	44%
5.13	95%	Day 4 meeting	67%
5.14	92-96% [6]	Questionnaire 3	83%
**Section 6**			
6.1	77%	Questionnaire 3	33%
6.2	79%	Prioritization survey	56%

[1] When part of an earlier recommendation, 96% agreement was for all definitions related to ‘predictable’, and 89% agreement was for all related to ‘unpredictable’.

[2] "Number of consecutive days with no bleeding for each instance of no bleeding if daily data were collected" did not reach consensus at 66% in the Prioritization Survey after further revision that followed initial revision and discussion during the Day 4 meeting.
[3] 5.5a is a combination of two related parts of a recommendations, which both reached consensus during Questionnaire 3, 81% for one and 85% for the other.
[4] 5.5b is a combination of related parts of a recommendations, which all reached consensus during Questionnaire 3 ranging from 81% to 89% (i.e., 89%, 81%, 85%, 85%).
[5] The definition of prolonged bleeding differed between the Data Working Group and Analysis Working Group, reflecting a difference between the International Federation of Gynecology and Obstetrics (FIGO) and the American College of Obstetricians and Gynecologists (ACOG). In Questionniare 3, "more than 8 days" (per FIGO) had 69% agreement, and "more than 7 days" had 85% agreement. To confirm this decision, experts completed a one-question survey about prolonged bleeding, and a majority agreed "more than 7 days" should be used for prolonged bleeding in all recommendations.
[6] 5.14 is a combination of two related recommendations from different working groups, which both reached consensus during Questionnaire 3, 92% for one and 96% for the other.
